# Designing a Standardised Referral Proforma From Primary Care to a Community Treatment Team

**DOI:** 10.1192/bjo.2026.11552

**Published:** 2026-06-30

**Authors:** Ayman Darwich, Satnam Goyal

**Affiliations:** Cumbria Northumberland Tyne and Wear NHS Trust, Newcastle upon Tyne, United Kingdom

## Abstract

**Aims:**

Patients are referred into the Community Treatment Team (CTT) on a daily basis. Referrals are discussed in a daily MDT and a decision is made on whether the patient is accepted into the service for a formal assessment and provision of care, signposted to a more suitable service, or discharged with advice to the referrer.

Our service currently does not have a standardised format for referrals, and variations in information provided often result in delays pending further information gathering/triage.

The aim of this project is to standardise referrals from Primary Care to the CTT by designing a proforma that will be circulated to GP surgeries. In order to justify our intervention, we first quantified the magnitude of the burden using the current referral system by reviewing referrals from a high volume supplier of referrals, identifying the cases that required further triage and quantifying the amount of delay caused.

**Methods:**

Retrospective review of all referrals from a single GP surgery over the month of October 2025. Original referral and MDT outcome obtained from Electronic Patient Records. Data collected on anonymised spreadsheet. Data analysis and graphical representations performed on MS excel.

**Results:**

Total 22 referrals. Sources of the referrals were 68% from GPs, 23% from Primary Care Psychology, 4.5% from EIP and 4.5% from inpatient services. It took on average 2.1 days (0-6 days) for the MDT to review referrals.

Outcome of referrals: 37% of referrals were accepted into the service, 27% were discharged with advice/signposting, 18% required further triage, 9% were referred onwards to relevant services and 9% were listed for discussion with Primary Care Psychology.

The common reasons for requiring triage were unclear risk (50%), unclear consent status (12.5%), unclear patient expectations (12.5%), unclear substance misuse (12.5%) and unclear current mental state (12.5%). Of those requiring triage, 75% were discharged post triage and 25% were accepted for full assessment. Triaging added an extra 4-15 days to a final MDT outcome.

**Conclusion:**

The results demonstrated that 18% of referrals were missing crucial information for a streamlined MDT outcome, and as a result causing delays for patients and additional burden on a stretched service. We have designed a referral proforma, based on the commonly used SBAR format, that includes prompts for risks, consent status and patient expectations. The new referral proforma is now being piloted in a selected number of GP surgeries and the impact will be re-measured using the same metrics as above.

